# The Use of Wireless, Smartphone App–Assisted Home Blood Pressure Monitoring Among Hypertensive Patients in Singapore: Pilot Randomized Controlled Trial

**DOI:** 10.2196/13153

**Published:** 2019-05-28

**Authors:** Eui Whan Moon, Ngiap Chuan Tan, John Carson Allen, Tazeen Hasan Jafar

**Affiliations:** 1 Programme in Health Services and Systems Research Duke-NUS Medical School Singapore Singapore; 2 SingHealth Polyclinics Singapore Singapore; 3 Centre for Quantitative Medicine, Office of Clinical Sciences Duke-NUS Medical School Singapore Singapore

**Keywords:** mHealth, smartphone, hypertension, home blood pressure monitoring, self blood pressure monitoring, health informatics, data collection methods, personal health records

## Abstract

**Background:**

Reliable home blood pressure monitoring (HBPM) is essential to effective hypertension management; however, manual recording is subject to underreporting and inaccuracies. Mobile health technologies hold great potential as HBPM tools, but the fidelity of a smartphone app in HBPM has not been adequately assessed.

**Objective:**

The primary aim of the trial was to compare the fidelity of a smartphone app to that of a handwritten logbook in making HBPM data available to clinicians at follow-up visits. Fidelity was defined as the percentage of scheduled blood pressure (BP) recordings over a 3-week period that were properly recorded and reported to the clinic. The secondary aims were to investigate patient factors associated with HBPM fidelity and to explore the effect of time on the fidelity.

**Methods:**

A 2-arm, parallel, unblinded, randomized controlled pilot trial was conducted in a government polyclinic in Singapore. Hypertensive adults, aged 40 to 70 years, who were on antihypertensive medication and owned a smartphone were recruited and randomized by a computer-generated randomization schedule to 3 weeks of either semiautomated HBPM utilizing a Bluetooth-enabled BP monitor and a smartphone app or a fully manual process utilizing a conventional handwritten logbook. The primary outcome was home BP recording fidelity.

**Results:**

Of the 80 patients randomized, 79 (smartphone app: 38 and logbook: 41) were included in the final analysis. Although fidelity was higher among the app users, it did not differ significantly between study arms (smartphone app: 66.7% and logbook: 52.4%; *P*=.21). Chinese and Indian ethnicities were associated with higher fidelity (absolute percent and 95% CI) by 35.6% (4.27 to 66.9) and 45.0% (8.69 to 81.3), respectively, in comparison with other ethnicities (*P*=.03); longer smartphone ownership increased fidelity on an average of 10.5% (0.83 to 20.2) per year (*P*=.03); the number of apps on the smartphone decreased fidelity at a rate of −0.32% (−0.58 to −0.05) per app (*P*=.02); years of hypertension morbidity increased fidelity at a rate of 1.56% (0.03 to 3.09) per year (*P*=.046); and the number of people working in the household decreased fidelity at a rate of −8.18% (−16.3 to −0.08) per additional working person (*P*=.048). The fidelity of the app was significantly higher in the first week (64.4%) than the second (55.1%, *P*=.001) and third (58.2%, *P*=.03) weeks of monitoring.

**Conclusions:**

Amid the increasing integration of health technologies into clinical practice, our study demonstrates the feasibility of smartphone app–assisted HBPM in hypertensive adults of Singapore. Our pilot study found no statistically significant difference in mean BP recording fidelity between a smartphone app and conventional handwritten logbook. However, the small sample size precludes definitive conclusions and highlights the need for a larger, adequately powered trial.

**Trial Registration:**

ClinicalTrials.gov NCT03209024; https://clinicaltrials.gov/ct2/show/NCT03209024 (Archived by WebCite at http://www.webcitation.org/78EVWBg0T)

## Introduction

### Background

Hypertension is the leading attributable risk factor for cardiovascular disease and death, globally [1]. The 2 blood pressure (BP) monitoring methods commonly used in primary care settings are home BP monitoring (HBPM) and office-based monitoring. HBPM is superior to office-based measurements as a predictor of cardiovascular disease and all-cause mortality [2]. Furthermore, the prognostic value of HBPM improves with the number of home BP measurements that patients are able to provide to their health care providers [3], thus emphasizing the importance of having a reliable means of collecting and reporting home BP data at each office visit. A systematic review and meta-analysis of 37 randomized controlled trials (RCTs) comparing HBPM with office BP measurements for effectiveness in reducing BP showed HBPM to be more effective in achieving BP control [4]. Nonetheless, at least one-third of known hypertensive patients in Singapore do not have adequate BP control per conventionally recommended levels [5]. This is especially problematic among the elderly in whom the prevalence of hypertension (73.9%) is higher, as is the rate of uncontrolled BP (75.9%) [6]. The lack of reliability in the conventional HBPM method could be an important contributing factor in the failure to achieve effective BP control and cardiovascular risk reduction in these patients. The shortcomings of conventional HBPM using handwritten logbooks are well known and include inaccuracies, underreporting of data [7], and failure to bring logbooks to clinic visits [8]. The purpose of HBPM is undermined and the value of reported measurements is diminished without an effective means of making accurate home BP readings available to clinicians. It is these considerations that motivate and necessitate the exploration of more reliable methods of communicating home BP values to health care providers.

### Mobile Health

Mobile health (mHealth) technology has been increasingly evaluated for chronic disease management. mHealth is broadly defined as any health care practice supported by mobile devices and their functionalities [9]. With the widespread use of smartphones in recent years, mobile apps have gained attention as an mHealth modality [10]. A content analysis of the top 107 apps for hypertension management showed that 72% include a tracking function for BP values [11]. This simple feature, when coupled with the wireless data transfer capabilities of Bluetooth-enabled BP monitors, would allow a mobile app to function as an electronic logbook that is more convenient to use and more readily accessible to clinicians than a handwritten log. Singapore, with a 91% smartphone penetration rate, holds favorable conditions to utilize a wireless platform in a clinical setting [12]. However, there are relatively few studies in the literature that present a quantitative comparison between a smartphone app and manual logbook in terms of their respective reliability as a recording tool for HBPM by patients. In addition, because operating technological devices is highly user-dependent, specific patient factors associated with the effectiveness of smartphone app–assisted HBPM need to be explored.

### Aims of This Study

Our pilot study aimed to begin addressing the abovementioned knowledge gaps by assessing whether there is any benefit in using mHealth technology (smartphone app) to store home BP values compared with using handwritten logbooks in terms of making these records available at clinic visits. The primary aim of our RCT was to compare the home BP recording fidelity over a 3-week period using a smartphone app versus a handwritten logbook in the Singaporean hypertensive patient population. Fidelity was defined as the percentage of scheduled home BP readings that are compliant with the HBPM regimen and are successfully reported at the follow-up visit. The null hypothesis postulated no difference in BP recording fidelity between a smartphone app and handwritten logbook. As there was no a priori basis for postulating greater fidelity with the app, the null hypothesis was tested against a 2-sided alternative, although the desired outcome was higher recording fidelity with the smartphone app. Secondary aims were (1) to explore associations between participant characteristics and the recording fidelity within each study arm and (2) to explore the effect of time on the weekly recording fidelity in each study arm. With detailed participant demographic data as well as HBPM records obtained via a logbook and the app, we compared the fidelity of the 2 home BP recording modalities, identified patient characteristics associated with higher fidelity for each monitoring method, described the attenuation pattern of fidelity in each study arm, and compared the fidelity of the 2 recording modalities on a weekly interval.

## Methods

### Trial Design

This study was an open-label, parallel group RCT of 2 study arms with a 1:1 allocation ratio.

### Ethics Approval and Trial Registration

The study protocol was approved by the SingHealth Centralised Institutional Review Board (reference #2017/2014) and registered under ClinicalTrials.gov (NCT03209024).

### Participants

#### Setting

Participants were recruited from Pasir Ris Polyclinic, a public primary care clinic serving the multi-ethnic population of a district in Singapore composed of approximately 143,000 residents.

#### Inclusion and Exclusion Criteria

Singaporean citizens or permanent residents aged between 40 and 70 years, visiting Pasir Ris Polyclinic for at least 1 year, diagnosed with essential hypertension and taking at least 1 antihypertensive medication, owning a compatible smartphone, and able to communicate in English were eligible.

Patients with cardiac arrhythmia, end-stage renal disease, cancer, history of stroke or myocardial infarction, or any other physical or mental disability that would prevent self-monitoring of BP at home were excluded. Other exclusions were arm circumference exceeding the cuff size, extensive travel overseas during the study period, working night shifts, or participation in another clinical trial.

### Screening and Recruitment

Patients were enrolled in the study via convenience sampling. Patients in the polyclinic waiting area were approached, a brief explanation of the study was provided, and prescreening questions were administered. Interested patients were subsequently screened for eligibility based on the study inclusion/exclusion criteria, and written informed consent was obtained from eligible patients. The informed consent procedure included an explanation of the purpose of the study, the study procedures and visit schedule, participants’ responsibilities and rights, and confidentiality of medical records.

### Randomization and Allocation Concealment

A computer-generated treatment allocation sequence accommodating 80 patients was generated by the study statistician (JCA) using permuted block randomization with blocks of size 6 and one block of size 8. Sequentially numbered, opaque, sealed envelopes (SNOSEs) were prepared, with each envelope containing a treatment group assignment. The primary investigator enrolled all patients, whereas allocation concealment and sequential dispensing of envelopes were enforced by an on-site research coordinator. Patients were randomly assigned to either the smartphone app or the handwritten logbook study groups.

### Home Blood Pressure Monitoring

#### Home Blood Pressure Monitoring Methods

Smartphone app–assisted HBPM was performed using the Bluetooth-enabled Omron HEM7280T BP monitor (Textbox 1) to wirelessly record BP values onto the Omron Connect app (Textbox 2), which was available free of charge on both Google Play store and Apple App Store and did not undergo major updates during the evaluation process. In brief, this was a semiautomated process that required patients to refresh the app’s home screen upon completion of a BP measurement to initiate the transfer of the reading from the HEM7280T BP monitor into the app’s electronic log. Logbook HBPM was performed by reading the BP values displayed on the BP monitor and recording them into a physical logbook. Sample screenshots of the Omron Connect app on iOS and Android platforms, as well as a sample image of the manual HBPM logbook, can be found in Multimedia Appendices 1,2, and 3, respectively. At the baseline visit, all the participants were instructed on how to properly record BP values using the HBPM method to which they were assigned, and they were given the opportunity to practice this process under supervision. All participants were provided with a phone number for technical support should they require any help troubleshooting errors with the app or BP monitor.

**Study device (Omron HEM7280T) specifications.**

Device Equivalent

- M6 AC (HEM-7322-E)—validated by the European Society of Hypertension protocol

Mode

- Fully automatic, oscillometric blood pressure (BP) monitor

Wireless feature

- Bluetooth-enabled device
- BP data (ie, systolic pressure, diastolic pressure, pulse rate, and time of measurement) transfer onto a smartphone app

BP measurement range

- 0-299 mmHg

Accuracy

- +/−3 mmHg

Internal memory capacity

- 2 user settings; 100 readings per user

Upper arm cuff circumference

- 22-42 cm

**Study device (Omron Connect Smartphone App) specifications.**

Wireless feature

- Synchronization and storage of the user’s blood pressure (BP) data from Omron HEM7280T via Bluetooth

Data export

- Able to export the log of BP data (date, time, time zone, systolic BP, diastolic BP, pulse, irregular heartbeat, cuff wrap guide, and BP device model) as a comma-separated value file using email or other apps (eg, WhatsApp and iMessage)

Compatible devices

- Compatible with both iOS and Android operating systems

#### Home Blood Pressure Monitoring Regimen and Blood Pressure Measurement Technique

All participants received the same instructions on the home BP recording regimen and the correct BP measurement technique. The home BP recording regimen was based on guidelines and recommendations of the European Society of Hypertension (ESH) [13,14] and consisted of consecutive duplicate readings in the morning (06:00 to 09:00 hours) and evening (18:00 to 21:00 hours). The patients were asked to follow the recording regimen over a 3-week (21 days) study period for a total of 84 measurements. The instructions for the BP measurement technique were adapted from recommendations by the authoritative sources [14-17].

### Data Collection

The Baseline Data Questionnaire was administered to obtain information on the participants’ sociodemographics, economic status, clinical characteristics, and exposure to technology. Baseline BP was recorded as the average of the last 2 of 3 consecutive BP measurements taken at 1-min intervals after 5 min of initial rest. The Hypertension Self-Care Profile questionnaire [18] is a validated tool to assess self-care behaviors in the domains of Behavior, Motivation, and Self-Efficacy. The version adapted to the Singaporean population was administered with permission from the authors [19].

At the end of the 3-week HBPM period, participants returned to the clinic for a single (final) follow-up visit. During the visit, the electronic log on the app used by the smartphone app group was exported to the study database for further analysis; similarly, a copy of the logbooks from the patients in the logbook group was uploaded into the study database. For security precautions, all participant data were anonymized and stored in password-protected computers or in locked cabinets.

Upon completion of the study, each participant received a Singapore $30 grocery store voucher.

### Statistical Analysis

#### Outcome Measures

The primary outcome measure was home BP recording fidelity, defined a priori as the percentage of scheduled home BP readings over the 3-week HBPM regimen which was recorded, regimen compliant, and successfully reported at the final clinic visit.

A secondary outcome of this study was *time-independent fidelity* which loosens the definition of fidelity by modifying the strict recording time criteria for determining which readings will be considered *HBPM regimen compliant* to include in the calculation of fidelity: the timeframe for a measurement to be considered regimen compliant was expanded to 01:01 to 13:00 hours for morning readings and 13:01 to 01:00 hours for evening readings. This study’s home BP recording regimen and recording time criteria (specified above) is useful to standardize outcome measures in the research setting, but these timeframes are largely arbitrary and are not followed strictly in the clinical setting. The secondary outcome of *time-independent fidelity* would allow this study to explore whether the strict recording time criteria of the HBPM regimen has any influence on the fidelity for each study arm. Weekly fidelity calculated as the percentage of scheduled weekly readings (28), which was HBPM regimen compliant and reported at the final clinic visit, was also assessed as a secondary outcome.

#### Description of Analytic Models

All data analysis was performed using SAS University Edition. Statistical significance was at *P*<.05.

Our study was an RCT. In consideration of the modest departure of home BP recording fidelity from a Gaussian distribution, the primary comparison on fidelity between the study groups was tested using the Wilcoxon rank-sum test appropriate for 2 independent samples. The median difference was estimated by the Hodges-Lehmann shift parameter estimate. In the sensitivity analyses, mean fidelity was also compared using (1) the 2-sample *t* test and (2) a general linear mixed-model, repeated-measures analysis to adjust the treatment group comparison for possible confounders of age, gender, and baseline systolic and diastolic BP, as well as to assess the effects of follow-up time (week) and time × treatment group interaction. In the latter statistical analysis, participants were included as random effects and time as a repeated-measures fixed effect within participants. The same sensitivity analyses were also performed on the outcomes defined under the *time-independent fidelity* between the 2 study arms. The data from the sole patient who withdrew from the study were omitted from the analyses.

The same general linear mixed-model was also used to assess the effect of time on weekly home BP recording fidelity in each study arm. Comparisons among weekly fidelities in the context of the mixed-model are analogous to paired *t* tests based on within-participant differences. A subgroup analysis on elderly patients (60≤age<70) was also performed using the same statistical method.

To investigate associations between participant baseline characteristics and home BP recording fidelity and to assess the predictive potential, a univariate and multivariate analysis of covariance was performed within each study arm. Baseline variables exhibiting significance at *P*<.20 in the univariate analysis were included in a stepwise multiple linear regression analysis to identify possible predictors of fidelity. Variables specific to exposure to technology were included in the analysis of the smartphone app arm but were excluded from the analysis of the logbook arm. From the variables selected in the stepwise analysis, only those significant at *P* ≤.10 were retained in the final model as potential predictors of home BP recording fidelity.

#### Sample Size Calculation

On the basis of a 2-sided 2-sample *t* test at alpha=.05, a sample size of n=35 patients per study arm was calculated to provide 80% power to detect an effect size (ES, Cohen *d*) of 0.6, where 0.5 is generally considered a *medium* ES. Overall, 80 participants were recruited anticipating a 10% withdrawal rate.

## Results

### Participant Flow and Baseline Characteristics

**Figure 1:**
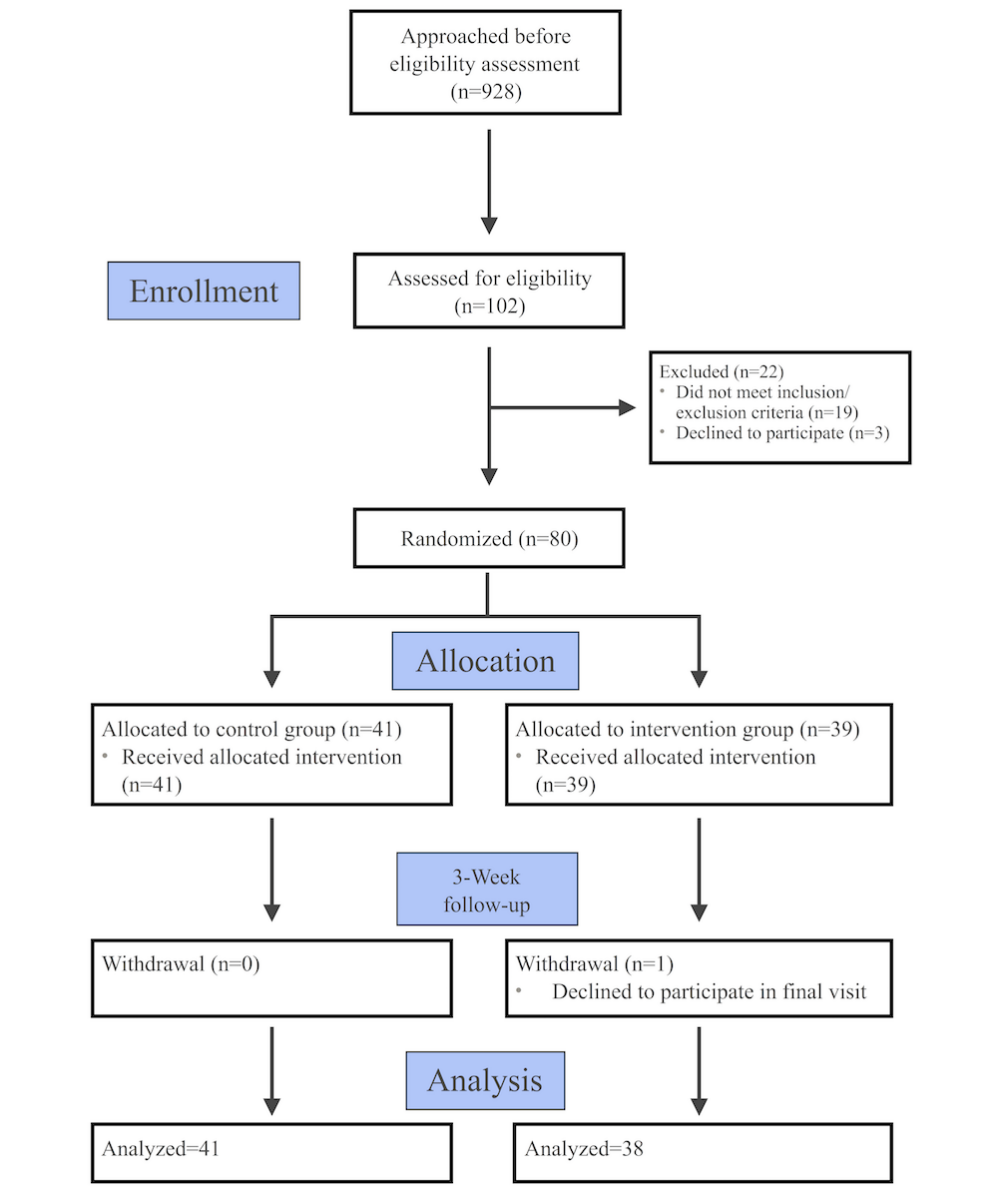
Consolidated Standards of Reporting Trials flow diagram.

A total of 928 patients were approached during the recruitment and follow-up period (03/15/2017–07/15/2017). As shown in the Consolidated Standards of Reporting Trials flow diagram (Figure 1), of the 102 patients undergoing screening, 83 were eligible for enrollment. Of those eligible, 80 were randomized. One patient randomized to the smartphone app arm was reallocated to the logbook arm at the baseline visit before commencing HBPM owing to unexplained smartphone incompatibility with the study app during the initial set-up process. One participant in the smartphone app arm withdrew from the study. In the smartphone app arm, 38 participants were included in the final analysis and 11 had home BP recording fidelity >80% at the end of the 3-week follow-up period. For the logbook arm, 41 were included in the analysis and 7 had home BP recording fidelity >80% at the end of 3 weeks.

The smartphone app and logbook arms were comparable in all baseline characteristics with the exception of systolic BP (SBP; Table 1). There was no evidence of SBP as a confounder.

### Home Blood Pressure Monitoring Fidelity

In the primary analysis on 79 participants (smartphone app: 38 and logbook: 41), higher median fidelity was achieved with the use of the smartphone app (66.7%) compared with the logbook (52.4%), although the difference was not statistically significant (Wilcoxon rank-sum, *P*=.21; Table 2). Similar results were obtained from comparisons of mean fidelity using the *t* test (*P*=.21) and the general linear mixed-model (*P*=.14).

In comparison with the primary outcome, the assessment of *time-independent fidelity* exhibited higher fidelity in both study arms, although the difference between arms was smaller and not statistically significant (*P*=.30; Table 2).

### Participant Characteristics Associated With Fidelity

For smartphone app–assisted HBPM, a multivariate analysis identified 5 independent baseline variables that exhibited statistically significant associations with fidelity: (1) The number of people working in the household was associated with decreased fidelity (absolute percent and 95% CI) by –8.18% (−16.3 to −0.08) per additional working person (*P*=.048); (2) Years of hypertension increased fidelity by 1.56% (0.03 to 3.09) per year (*P*=.046); (3) Years of current smartphone use increased fidelity on an average of 10.5% (0.83 to 20.2) per year of use (*P*=.03); (4) The number of apps on the current smartphone decreased fidelity by –0.32% (−0.58 to −0.05) per app (*P*=.02); (5) Mean fidelity had a significant association (*P*=.03) with ethnicity, with Chinese (n=24) and Indians (n=4) exhibiting higher fidelity by 35.6% (4.27 to 66.9) and 45.0% (8.69 to 81.3), respectively, in comparison with other ethnicities (n=3) composed of Pakistani, Ceylonese, and Eurasian ethnicities.

In the logbook arm, a multivariate analysis identified 3 independent baseline variables that exhibited significant associations with fidelity: (1) Working participants had significantly lower fidelity by 18.6% (1.51 to 35.8; *P*=.03) compared with nonworking participants; (2) Age was a positive factor with an average increase in fidelity by 1.21% (0.05 to 2.36) per year increase in age (*P*=.04); (3) For every additional child, fidelity declined by an average of –9.20% (–16.4 to –2.04; *P*=.01).

Self-care capacity, both in individual domains and the cumulative score of Hypertension Self-Care Profile, exhibited no evidence of association with fidelity.

**Table 1:** Baseline characteristics of study participants.

Variable				Total (N=79)		Logbook (n=41)		Smartphone app (n=38)		*P* value
**Sociodemographic characteristics**										
	Age, mean (SD)			56.3 (7.2)		56.1 (6.8)		56.5 (7.7)		.83
	**Gender, n (%)**									
		Male	37 (46.8)		19 (46.3)		18 (47.4)		>.99	
	**Ethnicity, n (%)**									
		Chinese	52 (65.8)		28 (68.3)		5 (12.2)		.92	
		Malay	13 (16.5)		6 (14.6)		7 (18.4)		.92	
		Indian	9 (11.4)		5 (12.2)		4 (10.5)		.92	
		Other	5 (6.3)		2 (4.9)		3 (7.9)		.92	
	**Highest level of education completed, n (%)**									
		Secondary or lower	34 (43.0)		14 (34.2)		20 (52.6)		.27	
		Postsecondary or Polytechnic diploma	28 (35.4)		17 (41.5)		11 (29.0)		.27	
		Degree or above	17 (21.5)		10 (24.4)		7 (18.4)		.27	
	**Marital status, n (%)**									
		Married	68 (86.1)		36 (87.8)		32 (84.2)		.75	
		Not married	11 (13.9)		5 (12.2)		6 (15.8)		.75	
	Number of children, mean (SD)			2.1 (1.1)		2.2 (1.1)		2.0 (1.1)		.49
**Economic status**										
	Currently working, n (%)			58 (73.4)		30 (73.2)		28 (73.7)		>.99
	**Ownership of house, n (%)**									
		Yes	75 (94.9)		40 (97.6)		35 (80.0)		.61	
		On rent	1 (1.3)		0 (0)		1 (2.6)		.61	
		Office accommodation	1 (1.3)		0 (0)		1 (2.6)		.61	
		Owned by relative or parents	2 (2.5)		1 (2.4)		1 (2.6)		.61	
	**Type of housing, n (%)**									
		Public housing (<5 rooms)	20 (25.3)		10 (24.4)		10 (26.3)		.77	
		Public housing (5+ rooms or HUDC^a^ or executive flat or studio)	48 (60.8)		24 (58.5)		24 (63.2)		.77	
		Private property	11 (13.9)		7 (17.1)		4 (10.5)		.77	
	Number of people in the house, mean (SD)			3.9 (1.6)		3.9 (1.6)		4.1 (1.4)		.56
	Number of people working in the household, mean (SD)			2.2 (1.1)		2.2 (1.1)		2.1 (1.0)		.79
	**Gross average monthly income of household, n (%)**									
		Below Singapore $8000	45 (57.0)		24 (58.5)		21 (55.3)		.82	
		Singapore $8000 and above	34 (43.0)		17 (41.5)		17 (44.7)		.82	
**Exposure to technology**										
	Number of personal electronic devices, mean (SD)			3.5 (1.8)		3.5 (1.8)		3.1 (1.6)		.27
	Regular use of computer, n (%)			59 (74.7)		29 (70.7)		30 (79.0)		.45
	Phone OS^b^, Apple, n (%)			44 (55.7)		23 (56.1)		21 (55.3)		>.99
	Years of smartphone use, mean (SD)			8.7 (3.3)		8.7 (3.3)		7.8 (3.2)		.23
	Years of current smartphone use, mean (SD)			1.8 (1.3)		1.8 (1.3)		1.8 (0.9)		.95
	Number of apps on current smartphone, mean (SD)			84.8 (54.9)		84.8 (54.9)		68.7 (34.1)		.12
	Hours of smartphone use per day, mean (SD)			4.7 (3.4)		4.7 (3.7)		4.4 (3.7)		.77
**Clinical characteristics**										
	Use of personal BP^c^ monitor at home, n (%)			72 (91.1)		37 (90.2)		35 (92.1)		>.99
	Years of personal BP monitor use, mean (SD)			4.8 (4.0)		4.8 (4.0)		4.6 (3.4)		.82
	Baseline systolic BP, mean (SD)			131.4 (15.5)		127.2 (17.1)		135.9 (12.1)		.01
	Baseline diastolic BP, mean (SD)			82.8 (9.9)		81.8 (11.1)		84.0 (8.5)		.33
	Years of hypertension, mean (SD)			8.0 (6.1)		8.3 (6.9)		7.7 (5.1)		.67
	Number of antihypertensive drugs, mean (SD)			1.6 (1.2)		1.6 (1.2)		1.6 (0.6)		.88
	Diabetes, n (%)			39 (49.4)		18 (43.9)		21 (55.3)		.37
	Hypertension Self-Care Profile—Behavior, mean (SD)			46.2 (8.5)		45.5 (9.14)		47.0 (7.79)		.43
	Hypertension Self-Care Profile—Motivation, mean (SD)			34.5 (9.2)		35.3 (9.24)		33.5 (9.11)		.38
	Hypertension Self-Care Profile—Self-Efficacy, mean (SD)			38.0 (9.4)		37.9 (10.3)		38.1 (8.49)		.91
	Hypertension Self Care Profile—Cumulative, mean (SD)			119.0 (22.9)		119.3 (25.6)		118.7 (20.1)		.90

^a^HUDC: Housing and Urban Development Company.

^b^OS: operating system.

^c^BP: blood pressure.

**Table 2:** Comparison of home blood pressure recording fidelity in all participants

Outcome measures and parameter		Smartphone app (n=38)	Logbook (n=41)	Difference (95% CI)	*P* value
**Fidelity^**a**^**					
	Median (IQR^b^)	66.7 (32.1 to 83.3)	52.4 (29.8 to 72.6)	8.33^c^ (−4.76 to 22.6)	.21^d^
	Mean (SD)	58.6 (29.2)	50.7 (27.2)	7.95 (−4.67 to 20.6)	.21^e^
	LS mean^f^ (RMSE^g^)	59.2 (28.3)	49.8 (28.3)	9.43 (−3.15 to 22.0)	.14^h^
**Time-independent fidelity**					
	Median (IQR)	88.7 (57.1 to 97.6)	84.5 (56.0 to 96.4)	2.38^c^ (−3.57 to 11.9)	.30^d^
	Mean (SD)	78.4 (22.4)	73.2 (27.4)	5.21 (−6.07 to 16.5)	.36^e^
	LS mean (RMSE)	78.8 (26.2)	72.8 (26.2)	6.01 (−5.36 to 17.4)	.30^h^

^a^Fidelity is defined as the percentage of specified home blood pressure readings over the 3-week home blood pressure monitoring regimen which was recorded, regimen compliant, and successfully reported at the final clinic visit.

^b^IQR: interquartile range.

^c^Hodges-Lehmann shift parameter estimate (median difference).

^d^Wilcoxon rank-sum test.

^e^2-sample *t* test.

^f^LS mean: least-squares mean.

^g^RMSE: root mean square error.

^h^Mixed-model longitudinal analysis of variance (ANOVA) adjusting for age, study arm, age by study arm interaction, and baseline systolic and diastolic blood pressures.

### Fidelity by Study Week

**Figure 2:**
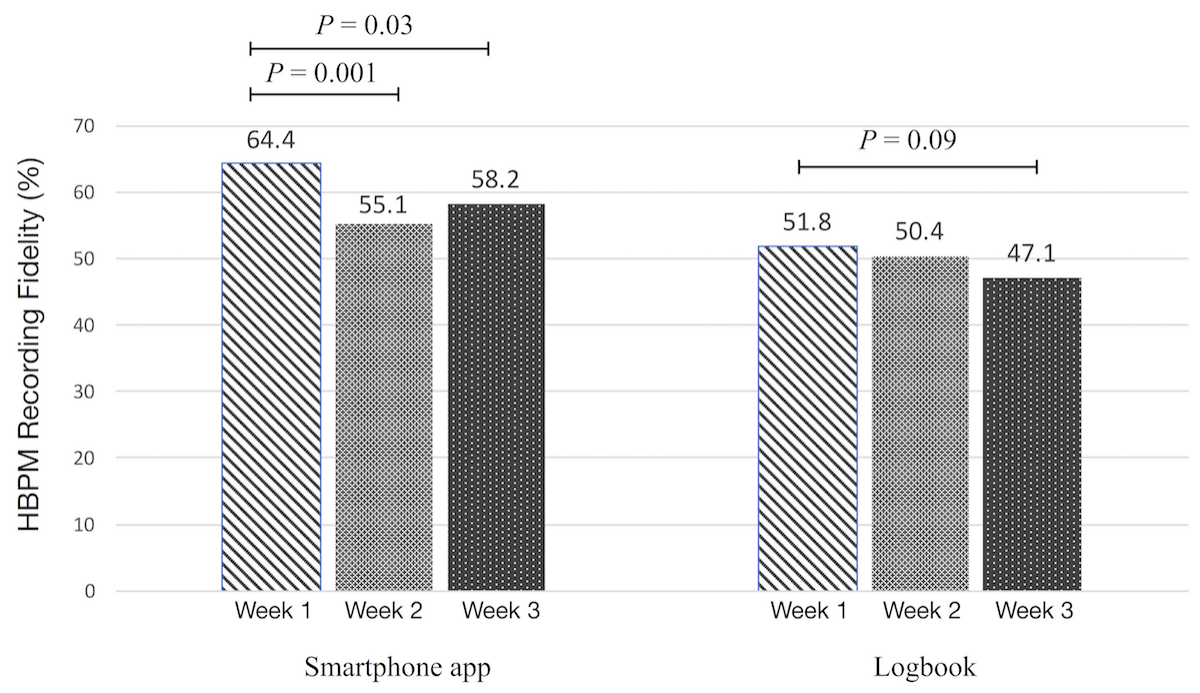
Weekly fidelity trend over 3 weeks in the smartphone app and logbook arms. HBPM: home blood pressure monitoring.

In assessing the weekly fidelity trend over the 3-week period within each study arm, fidelity was highest in the first week and lower in subsequent weeks (Figure 2). This difference was most pronounced among the app users, whose fidelity in the first week (64.4%) was significantly higher than that of the second (55.1%; *P*=.001) and third (58.2%; *P*=.03) weeks of monitoring. Though a similar trend was seen in the logbook arm, no change in fidelity among weeks was significant.

The participants using the smartphone app had higher weekly fidelity than their counterparts using the logbook in the first week, with the least-squares mean difference approaching significance (*P*=.06). Although mean fidelity for the smartphone app arm continued to outperform the logbook arm in the second and third weeks, these differences were less pronounced (Table 3).

**Table 3:** Comparison of home blood pressure recording fidelity by study weeks.

Parameter and week		Fidelity^a^						
		Smartphone app		Logbook		Difference (95% CI)	*P* value	
**Week 1; smartphone app (n=38) and logbook (n=41)**								
	LS mean^b^ (RMSE^c^)		64.4 (28.3)		51.8 (28.3)	12.6 (−0.63 to 25.8)		.06
	Median (IQR^d^)		64.3 (42.9 to 92.9)		53.6 (28.6 to 75.0)	14.3 (−3.57 to 25.0)		.12
**Week 2; smartphone app (n=38) and logbook (n=41)**								
	LS mean (RMSE)		55.1 (28.3)		50.4 (28.3)	4.68 (−8.54 to 17.9)		.49
	Median (IQR)		53.6 (21.4 to 85.7)		50.0 (32.1 to71.4)	3.57 (−10.7 to 17.9)		.62
**Week 3; smartphone app (n=38) and logbook (n=41)**								
	LS mean (RMSE)		58.2 (28.3)		47.1 (28.3)	11.0 (−2.22 to 24.2)		.10
	Median (IQR)		64.3 (35.7 to 78.6)		50.0 (28.6 to 71.4)	10.7 (−3.57 to 25.0)		.16

^a^Fidelity is defined as the percentage of specified weekly home blood pressure readings which was recorded, regimen compliant, and successfully reported at the final clinic visit.

^b^LS mean: least-squares mean.

^c^RMSE: root mean square error.

^d^IQR: interquartile range.

### Subgroup Analysis in the Elderly

In a posthoc assessment of the elderly participants (60≤age<70), there was no significant difference in overall mean fidelity between app and logbook users (*P*=.10; Table 4). However, in the assessment of weekly recording fidelities, app users exhibited significantly higher fidelity (79.0%) than logbook users (58.8%) during the first week of monitoring (*P*=.048; Table 5).

**Table 4:** Comparison of home blood pressure recording fidelity by age.

Parameter and age		Fidelity^a^			
		Smartphone app	Logbook	Difference (95% CI)	*P* value
**40<age<50; smartphone app (n=8); logbook (n=6)**					
	LS mean^b^ (RMSE^c^)	49.5 (35.8)	51.7 (35.8)	−2.26 (−46.7 to 42.2)	.91
	Median (IQR^d^)	58.9 (31.5 to 79.8)	51.8 (20.2 to 86.9)	0.595 (−46.4 to 50.0)	>.99
**50≤age<60; smartphone app (n=17); logbook (n=21)**					
	LS mean (RMSE)	48.3 (30.2)	44.8 (30.2)	3.49 (−16.5 to 23.5)	.72
	Median (IQR)	40.5 (25.0 to 72.6)	40.5 (20.2 to 66.7)	2.38 (−15.5 to 23.8)	.68
**60≤age<70; smartphone app (n=13) logbook (n=14)**					
	LS mean (RMSE)	74.9 (22.2)	59.0 (22.2)	15.9 (−3.13 to 35.0)	.10
	Median (IQR)	79.8 (71.4 to 92.9)	58.3 (45.2 to 72.6)	18.4 (−1.19 to 36.9)	.07

^a^Fidelity is defined as the percentage of specified home blood pressure readings over the 3-week home blood pressure monitoring regimen which was recorded, regimen compliant, and successfully reported at the final clinic visit.

^b^LS mean: least-squares mean.

^c^RMSE: root mean square error.

^d^IQR: interquartile range.

**Table 5:** Comparison of home blood pressure recording fidelity in the elderly subgroup (60≤age<70).

Parameter and week		Fidelity^a^			
		Smartphone app	Logbook	Difference (95% CI)	*P* value
**Week 1; smartphone app (n=13) and logbook (n=14)**					
	LS mean^b^ (RMSE^c^)	79.0 (22.2)	58.8 (22.2)	20.1 (0.22 to 40.0)	.048
	Median (IQR^d^)	92.9 (64.3 to 96.4)	62.5 (50.0 to 78.6)	21.4 (3.57 to 39.3)	.04
**Week 2; smartphone app (n=13) and logbook (n=14)**					
	LS mean (RMSE)	73.2 (22.2)	62.4 (22.2)	10.8 (–9.13 to 30.7)	.28
	Median (IQR)	82.1 (50.0 to 92.9)	64.3 (50.0 to 78.6)	14.3 (–7.14 to 32.1)	.18
**Week 3; smartphone app (n=13) and logbook (n=14)**					
	LS mean (RMSE)	72.7 (22.2)	55.8 (22.2)	16.9 (–3.04 to 36.8)	.10
	Median (IQR)	71.4 (60.7 to 85.7)	53.6 (42.9 to 71.4)	17.9 (0.00 to 35.7)	.08

^a^Fidelity is defined as the percentage of specified home blood pressure readings over the 3-week home blood pressure monitoring regimen which was recorded, regimen compliant, and successfully reported at the final clinic visit.

^b^LS mean: least-squares mean.

^c^RMSE: root mean square error.

^d^IQR: interquartile range.

## Discussion

### Principal Findings

Our study compared the recording fidelity of an app-mediated electronic record versus a handwritten logbook in performing HBPM. Although higher fidelity was observed in the smartphone app arm compared with the logbook arm, indicating the potential for improved fidelity with the use of the app, statistical significance was not achieved. Meanwhile, the findings of our secondary aims have provided valuable information on the fidelity of the app-assisted method by identifying a unique set of potential predictors and describing an attenuation pattern over time. Finally, the post hoc subgroup analysis by age group showed significantly higher recording fidelity with the app versus the handwritten logbook in the elderly during week 1 of monitoring, suggesting that app-assisted HBPM is feasible across a wide range of ages. These findings have promising implications for the expanding use of mHealth technology in hypertension management and warrant further investigation in future studies.

There remains a scarcity of the literature addressing a direct comparison of home BP recording fidelity between app-mediated and handwritten methods. Although some studies do report outcome measures similar to fidelity as we define it (eg, level of adherence to a given HBPM regimen), heterogeneity in regimen frequency and duration make comparisons of study results difficult [3]. In addition to reliable recording fidelity, adequate patient support may also be important to achieving effective clinical outcomes. The findings from a recent systematic review and meta-analysis showed that HBPM alone did not have a higher association with BP lowering and BP control compared with no-home monitoring (usual care), whereas HBPM with additional patient support via feedback, education, and counselling demonstrated a significantly higher association [20]. This emphasizes the importance of future studies to further explore smartphone app use, not only as a high-fidelity recording tool for HBPM but also as a platform for delivering effective patient adherence support such as health information and reminders [21].

From the secondary aim of our study, we found that the participant factors associated with higher fidelity were not the same for the 2 HBPM modalities, suggesting that determinants of fidelity in manual recording of home BP are different from those in smartphone use. To our knowledge, this is the first study to identify potential predictors of fidelity for app-assisted HBPM, and this novel information could be utilized in future studies to develop a clinically useful predictive model that provides decision support for identifying patients most suitable for mHealth-based monitoring. Although determining the mechanism of how certain patient factors lead to higher HBPM fidelity is beyond the scope of this study, we do offer plausible explanations for these associations. Among app users, longer ownership of a smartphone may mean greater proficiency with one’s own device, thus improving the performance of app-mediated HBPM. The number of apps on the phone exhibited a negative association with fidelity, although we find the clinical relevance of this finding questionable as the impact of an additional app on fidelity was negligible. A longer duration of hypertension and fewer working household members were also associated with higher fidelity, perhaps because patients who have been hypertensive for many years may be more accustomed to following an HBPM regimen, whereas those who have more employed family members may have fewer people available at home to provide family support for hypertension management. For handwritten logbook-mediated HBPM, age was associated with fidelity whereas employment and number of children exhibited negative associations. This may reflect the difficulty in manually recording home BP values on a rigorous regimen while simultaneously being occupied with commitments at work or home. With advancing age and eventual retirement, adherence may improve as demands at home and work decrease.

The significant attenuation of fidelity from weeks 1 to 2 in the smartphone app arm may be explained in part by the duration of BP monitoring. The ESH guidelines specify a 7-day period of monitoring for its HBPM regimen [13,14], and prolonging this rigorous regimen beyond the recommended period of 1 week may have led to study fatigue among participants in the smartphone app arm.

The post hoc subgroup analysis by age suggested that the elderly perform HBPM better with the app than the manual logbook, especially during week 1 of monitoring. This finding cannot be regarded as conclusive but suggests that the elderly do not lack in the smartphone proficiency needed for recording home BP, which challenges the common preconception that mHealth-based HBPM may be too complicated for older patients.

The use of a 2-sample test in the primary analysis with no adjustment for potential confounders was appropriate. Of the 30 covariates measured at baseline, only one (SBP) resulted in a significant difference between study groups, which is consistent with the expected false positive error rate for 30 individual tests performed at the 5% significance level. Moreover, there was no evidence that SBP was associated with fidelity.

### Limitations and Strengths

This study has a number of limitations. First, as a pilot trial, the sample size was limited by time and available resources. As we had no knowledge of either the expected difference in mean fidelity or the population SD, the sample size calculation was based on a targeted Cohen ES of 0.6, which was the smallest feasible and realistically achievable ES, given the available resources and timeframe of the study. The observed ES for fidelity was 0.33, hence the study was obviously underpowered. Second, recruitment was limited to one polyclinic and the participants were exclusively current smartphone users, which could preclude the generalizability of the results to the greater hypertensive population in Singapore or around the world. Third, this was an unblinded study because participants were required to learn the procedures for their respective HBPM methods; investigators were not masked, but we attempted to reduce bias by defining our primary outcome measure and the primary statistical method a priori. Finally, the Hawthorne effect could not be precluded, and the magnitude of the effect on the 2 study arms may have differed. A future full-scale trial of longer duration may mitigate the Hawthorne effect as patients become accustomed to home BP recording over the course of several months or years.

With regard to the strengths of our study, to our knowledge, this is the first RCT to directly compare the recording fidelity of mHealth and the conventional logbook methods of HBPM within the multi-ethnic Southeast Asian population. In addition, although the HBPM regimen used in research settings can vary widely, the regimen used in our study was adopted from an established clinical guideline and recommendations by ESH, which contributes to the reproducibility of this study and standardizes its outcome to allow comparison with other studies that also use this well-established HBPM regimen.

### Future Directions

For the future full-scale trial to be sufficiently powered, the sample SD of approximately 28% and mean difference in fidelity of about 8% observed in this study can be used to ensure that a 2-arm study with n=175 participants per group would provide 80% power to detect an ES of 8/28 (approximately 0.30) at alpha=.05.

Although beyond the scope of this study, there remains a need to categorically quantify and compare the influence of over-reporting, underreporting, and falsifying data on the fidelity of logbook- and app-mediated HBPM to guide future app design to promote accuracy in data transfer from home to clinics. Moreover, other important clinical outcomes in hypertension management, such as the magnitude of BP lowering and proportion of BP control, must be assessed.

As with any novel health care implementation, the sustainability of app-mediated HBPM is another area that deserves the focus of future research. As intuition would suggest, health apps are not immune to attenuation of usage over time, which has direct implications on the home BP recording fidelity as was observed in our results as well. One study on the effect of the usage pattern of a health care app on user retention suggests the frequency of utilizing the self-monitoring function of the app—as opposed to other functions such as outpatient support service or medication functions—significantly increases the probability of the app to stay in use [22]. The authors of that study suggest that the benefit of achieving better health outcomes by modifying health behavior based on tracking one’s own health parameters is the mechanism behind how frequent utilization of the self-monitoring function of a health app promotes its sustained use. The self-monitoring function is at the core of app-mediated HBPM, therefore it is vital for future studies not only to describe attenuation patterns of app use over time but also to explore modifiable factors (including BP recording regimens and the ease of app user interface) that promote frequent self-monitoring, thereby improving the app’s retention rate.

### Conclusions

As health technologies continue to find an increasingly profound integration into clinical practice, our study makes a contribution to the sparsity of available knowledge on the fidelity of smartphone app–assisted HBPM. Although our pilot study did not find a significant difference in BP recording fidelity between app-assisted and logbook-mediated HBPM, our results have identified unique determinants of fidelity in the 2 recording methods of home BP, which has not been reported before, and have characterized the attenuation of fidelity in time. Our findings also suggest that app-assisted HBPM is viable across a wide age spectrum that includes the elderly.

## Appendix

Multimedia Appendix 1 Screenshot of Omron Connect app on iOS platform.

Multimedia Appendix 2 Screenshot of Omron Connect app on the Android platform.

Multimedia Appendix 3 Image of manual home blood pressure monitoring logbook.

Multimedia Appendix 4 CONSORT‐EHEALTH checklist (V 1.6.1).

## Abbreviations

BP: blood pressure
ES: effect size
ESH: European Society of Hypertension
HBPM: home blood pressure monitoring
mHealth: mobile health
RCT: randomized controlled trial
SBP: systolic blood pressure
SNOSE: sequentially numbered, opaque, sealed envelope
