# Supplementary figures and images for: The Use of Wireless, Smartphone App–Assisted Home Blood Pressure Monitoring Among Hypertensive Patients in Singapore: Pilot Randomized Controlled Trial
Source: JMIR Mhealth Uhealth. 2019 May 28;7(5):e13153. doi: 10.2196/13153 (PMC6658261; doi:10.2196/13153)

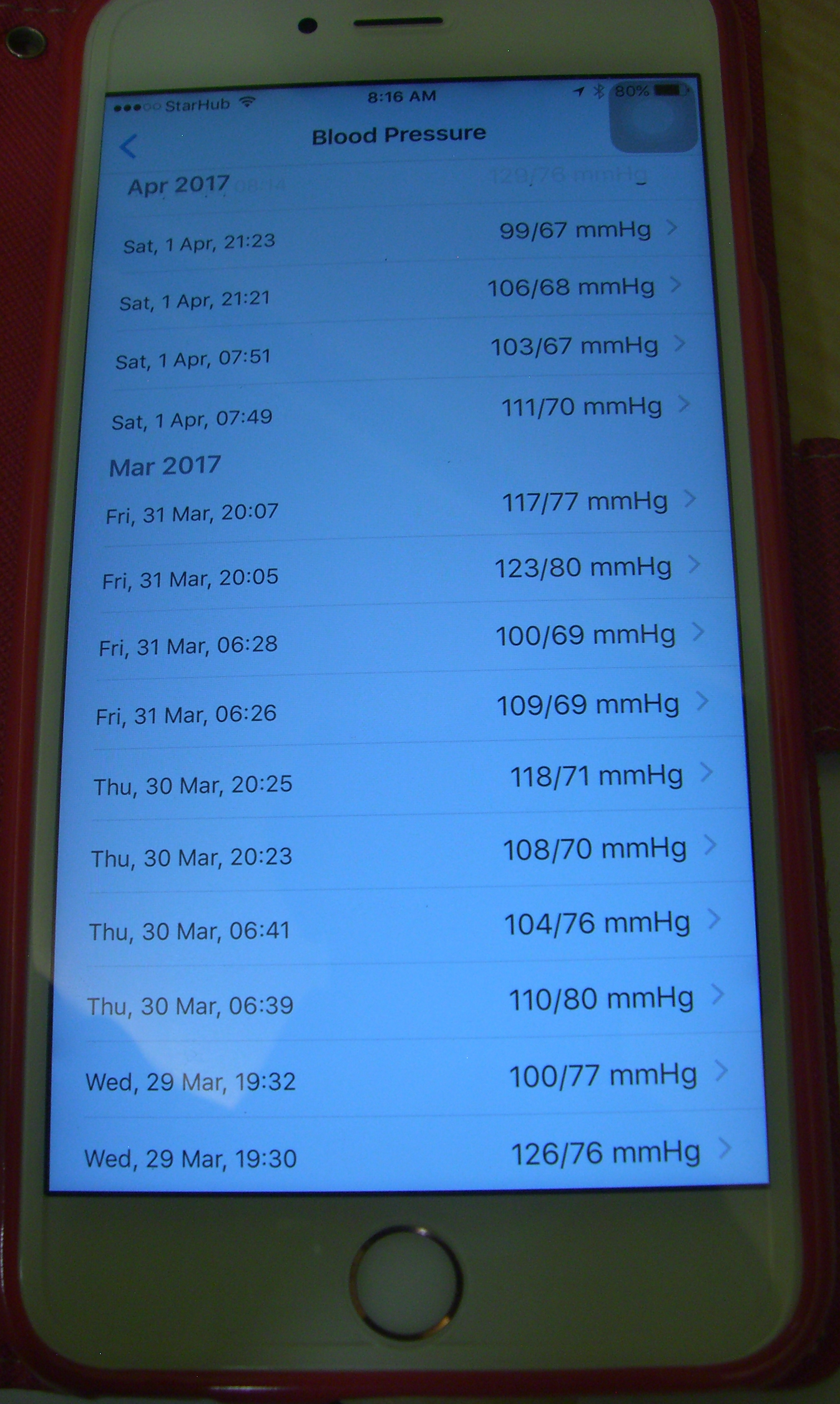

Supplement: Multimedia Appendix 1 [file mhealth_v7i5e13153_app1.png]

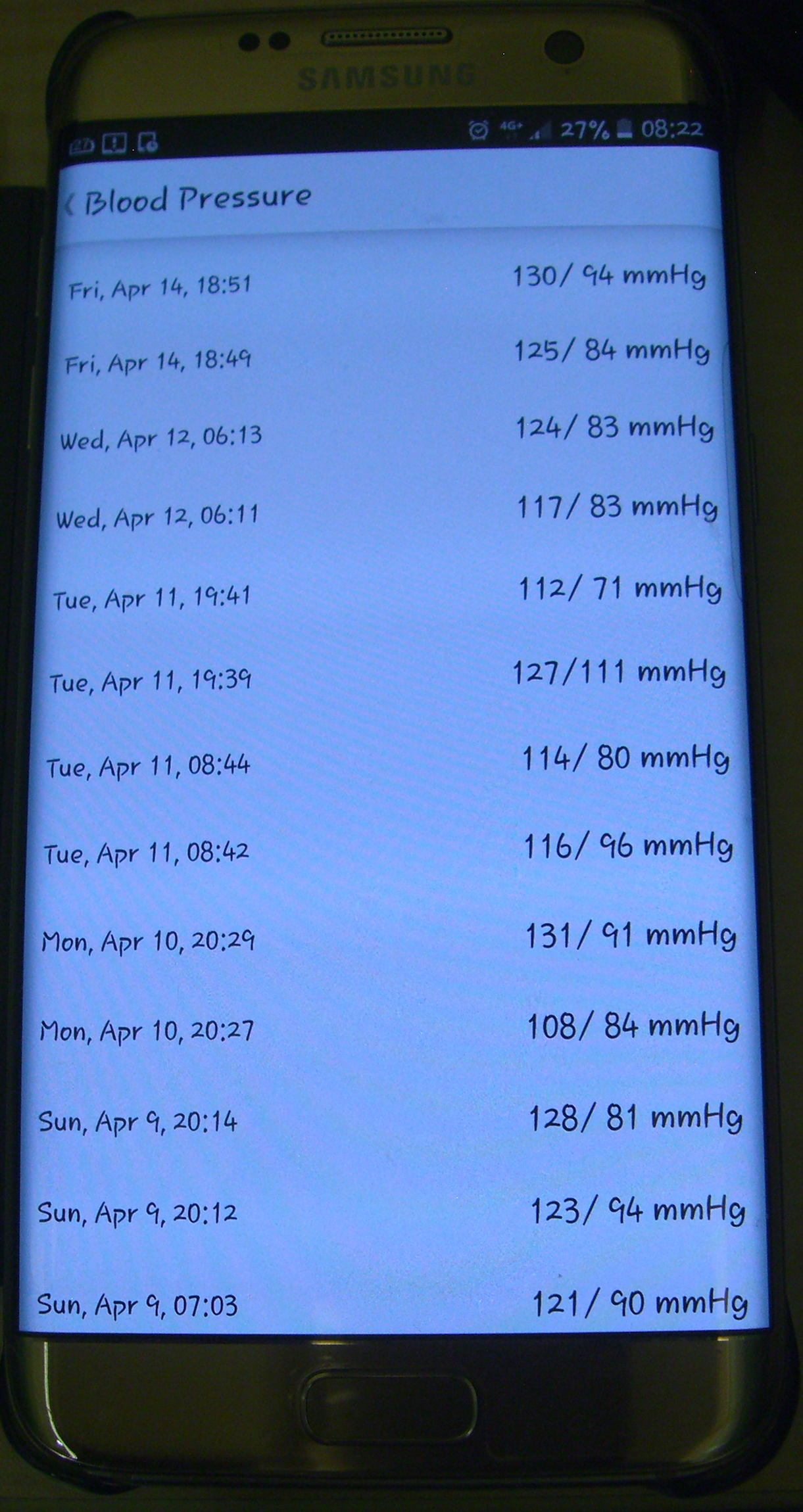

Supplement: Multimedia Appendix 2 [file mhealth_v7i5e13153_app2.png]

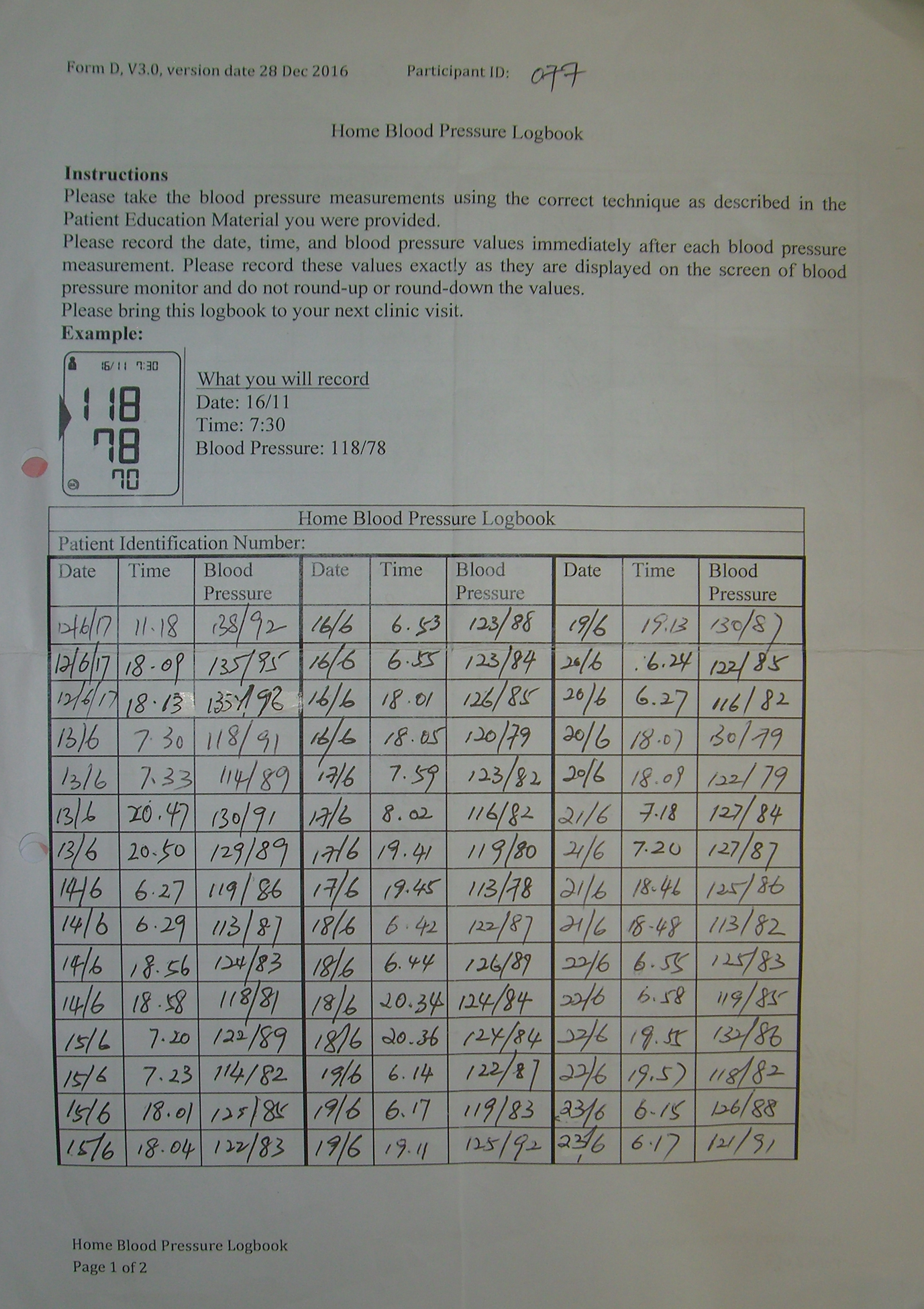

Supplement: Multimedia Appendix 3 [file mhealth_v7i5e13153_app3.png]
